# Basement Membrane Zone Collagens XV and XVIII/Proteoglycans Mediate Leukocyte Influx in Renal Ischemia/Reperfusion

**DOI:** 10.1371/journal.pone.0106732

**Published:** 2014-09-04

**Authors:** Azadeh Zaferani, Ditmer T. Talsma, Saleh Yazdani, Johanna W. A. M. Celie, Mari Aikio, Ritva Heljasvaara, Gerjan J. Navis, Taina Pihlajaniemi, Jacob van den Born

**Affiliations:** 1 Nephrology, University Medical Center Groningen, University of Groningen, Groningen, The Netherlands; 2 Molecular Cell Biology and Immunology, VU University Medical Center, Amsterdam, The Netherlands; 3 Oulu Center for Cell-Matrix Research, Biocenter Oulu and Faculty of Biochemistry and Molecular Medicine, University of Oulu, Oulu, Finland; University of Patras, Greece

## Abstract

Collagen type XV and XVIII are proteoglycans found in the basement membrane zones of endothelial and epithelial cells, and known for their cryptic anti-angiogenic domains named restin and endostatin, respectively. Mutations or deletions of these collagens are associated with eye, muscle and microvessel phenotypes. We now describe a novel role for these collagens, namely a supportive role in leukocyte recruitment. We subjected mice deficient in collagen XV or collagen XVIII, and their compound mutant, as well as the wild-type control mice to bilateral renal ischemia/reperfusion, and evaluated renal function, tubular injury, and neutrophil and macrophage influx at different time points after ischemia/reperfusion. Five days after ischemia/reperfusion, the collagen XV, collagen XVIII and the compound mutant mice showed diminished serum urea levels compared to wild-type mice (all p<0.05). Histology showed reduced tubular damage, and decreased inflammatory cell influx in all mutant mice, which were more pronounced in the compound mutant despite increased expression of MCP-1 and TNF-α in double mutant mice compared to wildtype mice. Both type XV and type XVIII collagen bear glycosaminoglycan side chains and an *in vitro* approach with recombinant collagen XVIII fragments with variable glycanation indicated a role for these side chains in leukocyte migration. Thus, basement membrane zone collagen/proteoglycan hybrids facilitate leukocyte influx and tubular damage after renal ischemia/reperfusion and might be potential intervention targets for the reduction of inflammation in this condition.

## Introduction

Extracellular matrix (ECM) forms a major part of the cell microenvironments and affects many cellular functions such as differentiation, proliferation and migration. Basement membranes (BM) represent a specialized ECM which forms a thin sheet-like structure around or below cells [1]. In the kidney all (micro)vessels and tubules are endowed with a subendothelial and subepithelial BM, respectively. The key proteins in the BMs include laminin, collagen IV, nidogens, agrin and perlecan, but they also contain numerous other components such as collagen XVIII, and in the adjacent fibrillar matrix also collagen XV [2], [3]. Collagens XV and XVIII assemble to homotrimers and are classified as the multiplexin group due to the presence of multiple non-collagenous interruptions in their central triple-helix. Both collagen XV and XVIII are also proteoglycans (PG). Collagen XVIII is a heparan sulfate PG (HSPG) while in collagen XV glycosaminoglycan (GAG) side chain composition is variable, in the kidney mainly HS [4]. Collagen XVIII occurs as three N-terminal variant isoforms, which are expressed in a tissue-specific manner [5], [6]. In the short isoform a trombospondin-1-like (Tsp1) sequence at the N-terminus precedes the central collagenous domain. The longest isoform also includes a domain of unknown function (DUF), as well as a frizzled domain FZC18, which is spliced out from the middle variant [7]. The C-terminal non-collagenous domain, present in all isoforms, contains a 20-kDa endostatin part which can be proteolytically cleaved and displays angiostatic properties *in vitro* and *in vivo* [7], [8]. Collagen XV also has an endostatin-like region in its non-collagenous C-terminal which is called restin [9]. Mutations in collagen XVIII in humans results in the Knobloch syndrome [10]. This syndrome is characterized by an ocular phenotype, which is also seen in mice lacking collagen XVIII [11]. There are no known collagen XV mutations in human, however mice deficient for collagen XV show skeletal myopathy, impaired cardiac function and defects in microvasculature of the heart and skin [12], [13]. During Drosophila embryonic development, the collagen XV/XVIII orthologue is involved in stabilizing morphogen gradients, most likely by their HS side chains [14], [15].

In the kidney, both collagen XV and XVIII are found in BMs, including Bowman’s capsule, glomerular, tubular and vascular BM and mesangial matrix [16], [17]. Previous ultrastructural analyses have revealed that collagen XV is associated with fibrillar collagen network adjacent to epithelial and endothelial BMs [4], [13]. Collagen XVIII, on the other hand, is an integral BM protein, its short isoform being the main form in endothelial BMs [7] and the sole form in the kidney tubular BM [16]. However, in the glomerular BM (GBM) more than one forms are present, the short being the main form in the endothelial side while the longer forms locate in the podocyte side of the GBM [16]. It has been shown that collagen XVIII is upregulated shortly after renal ischemia/reperfusion (I/R) and after renal transplantation, and is involved in glomerulonephritis [18]–[20]. In addition, we have shown that monocyte influx upon renal I/R is impaired in mice lacking the BM HSPGs perlecan and collagen XVIII [21].

Renal I/R injury is a process that always occurs after renal transplantation, as well as in acute kidney injury. Although the mechanisms of I/R injury are incompletely understood, the involvement of the innate immune system in I/R injury is well established. Upregulation of inflammatory chemokines, adhesion molecules, complement factors and ECM components upon I/R have been shown [21]–[23]. I/R is characterized by an initial phase of inflammation followed by a repair phase in which tubular cells start to proliferate and differentiate to repair tubular damage [24], [25]. The roles of vascular BM, and more specifically of the BM associated collagens XV and XVIII/HSPGs hybrid molecules in perivascular inflammatory cell recruitment and tissue injury are incompletely understood. Therefore, we investigated the role of collagen XV and XVIII in renal I/R injury and repair. Using mice mutant for collagen XVIII (*Col18a1^−/−^*), collagen XV (*Col15a1^−/−^*), and their compound mutants (*Col15a1^−/−^×Col18a1^−/−^*), in a bilateral renal I/R model, we evaluated renal function and histopathological changes at day 1, 5 and 10 after I/R.

## Materials and Methods

### Animals and Renal ischemia/reperfusion

Adult wild-type (WT), *Col18a1^−/−^* [11], *Col15a1^−/−^* [12], and compound mutant *Col15a1^−/−^×Col18a1^−/−^* male mice [26], all in C57BL/6 background and ranging from 10 to 18 weeks old, were used for the experiment. Bilateral renal warm I/R was performed under general anesthesia through a midline abdominal incision by closing the right and left renal pedicle with microaneurysm clamps for 25 minutes. After clamp removal, kidneys were visually checked for restoration of blood flow. The abdomen was closed, and mice received a subcutaneous injection of 50 µg/kg buprenorphin (Temgesic; Schering-Plough) for analgetic purposes. Four to seven mice per genetic group were sacrificed using carbon dioxide at selected timepoints at day 1, day 5 or day 10 after reperfusion. Sham-operated mice underwent the same procedure without clamping and were sacrificed using carbon dioxide 1 day after the surgery. Both kidneys were removed and preserved either snap-frozen, or formalin-fixed and paraffin-embedded. Blood samples were collected at the time of sacrification and serum urea was measured on a multi-test analyzer system (Roche Modular; F. Hoffmann-La Roche Ltd, Basel, Switzerland) at the central clinical laboratory of the University Medical Center Groningen. The animal experiments were approved by the National Animal Experiment Board in Finland.

### Histology and immunohistology

To determine the tubular damage, formalin-fixed and paraffin-embedded kidney sections (4 µm) were stained with periodic acid Schiff's (PAS) reagent. Tubules showing necrosis (defined by the loss of nuclei) were quantified in ten non-overlapping photographs (×400 magnification) in the outer medullary zone of both kidneys and expressed as a mean percentage of affected tubuli. For quantification of neutrophils, macrophages and MCP-1, acetone fixed cryosections (4 µm) were blocked with 5% normal goat serum, followed by incubation of rat anti-neutrophil (Serotec, Oxford, UK), rat anti-macrophage (F4/80; eBioscience, CA, USA) antibodies, and rabbit ant-mouse MCP-1 (FL-148; SantaCruz, Dallas, Texas, USA) respectively. Goat anti-rat IgG HRP (Jackson ImmunoResearch, Suffolk, UK) or goat anti-rabbit IgG HRP (DAKO, Glostrup, Denmark) followed by TSA tetramethylrhodamine signal amplification system (PerkinElmer LAS Inc., Boston, USA) was used to visualize the signals. Stained neutrophils and macrophages were counted in the outer medullary region of both kidney specimens from ten non-overlapping fields (×400 magnification). Collagen XVIII staining was done on acetone-fixed cryosections after blocking with 5% normal goat serum. Sections were incubated with a rabbit anti-mouse collagen XVIII NC11 antibody (kindly provided by Dr. T. Sasaki, Dept. Biochemistry and Molecular Biology, Oregon Health and Science University, Portland, OR, USA), followed by secondary FITC-conjugated goat anti-rabbit antibody (Southern Biotech, Alabama, USA). Collagen XV was visualized in acetone-fixed cryosections with a rabbit anti-mouse collagen XV antibody (Heljasvaara *et al*., unpublished) in a similar way. To detect VCAM-1 expression, cryosections were fixed with acetone and endogenous peroxidase was inactivated by 0.5% phenylhydrazine (Envision kit, DAKO, Glostrup, Denmark). Sections were blocked with 1% BSA, followed by sequential incubations with rat anti-VCAM-1 (Millipore, CA, USA), rabbit anti-rat IgG (DAKO, Glostrup, Denmark) and HRP-conjugated anti-rabbit IgG (Envision kit, DAKO, Glostrup, Denmark). The signal was visualized by AEC (Envision kit, DAKO, Glostrup, Denmark). Tubular expression of VCAM-1 was determined in the whole kidney at ×50 magnification using ImageJ 1.41 (Rasband, W.S., ImageJ, U.S. National Institutes of Health, Bethesda, Maryland, USA). Images of immunofluorescent staining were captured with confocal microscopy (Zeiss LSM 780, Jena, Germany) at the University Medical Center Groningen, Imaging and Microscopy Center (UMIC). A Leica DM 2000 LED (Leica microsystems BV., Rijswijk, the Netherlands) microscope equipped with a Leica DFC 450 camera was used for light microscopy.

### Gene transcript analysis

RNA was isolated from frozen tissue by Tissue Total RNA extraction kit ( Favorgen Biotech Corp, Vienna, Austria) according to the manufacturer protocol. RNA concentration and integrity were determined by spectrophotometry (Nanodrop Technologies, Wilmington, DE). For quantitative reverse transcription-polymerase chain reaction (qRT-PCR), total RNA was reverse transcribed using Qiagen reverse transcription kit (Venlo, the Netherlands) in accordance to the manufacturer's protocol. Subsequently, samples for qRT-PCR, consisting of 5 µl cDNA (1 ng/µl), 4,5 µl SYBR Green Supermix (BioRad, Veenendaal, The Netherlands) and 0.5 µl gene specific primer (0.5 mM), were pipetted into a 384 wells plate (Applied Biosystems, Foster City, CA). All reactions were performed in triplicate. The primers used for MCP-1: forward accagcagcaggtgtccc and reverse gcacagacctctctctcttgagctt, for TNF-α forward: catcttctcaaaattcgagtgacaa and reverse tgggagtagacaaggtacaaccc, and for VCAM forward: acccaaacagaggcagagtg and reverse cacttgagcaggtcaggttc were all purchased from Sigma. Amplification was performed using an ABI7900HT Thermal cycler (Applied Biosystems) with 40 thermal cycles of 95°C for 15 s, 57°C for 30 s and 72°C for 30 s. Data analysis was performed using science detection software 2.4 (Applied Biosystems). To determine differences in expression of gene of interest, Ct-values were normalized against mean Ct-values of ribosomal 36B4 housekeeping gene (forward: ggccaataaggtgccagct and reverse: tgatcagcccgaaggagaag) using ΔCt-method: ΔCt = Ct _gene of interest_ – Ct _mean 36B4_. Relative expression of gene of interest was calculated as 2^−(ΔCt)^.

### Expression and purification of recombinant collagen XVIII

The entire N-terminal non-collagenous domain of short collagen XVIII comprising the amino acid residues 1–325 and including Tsp-1 sequence was amplified by PCR from murine *Col18a1* cDNA(26) using the primers 5′-ctagata∧agcttctggggagatggcgcccaggtggcacctc-3′ (HindIII cleavage site) and 5′-tcattgt∧ctagactaa*tggtgatggtgatgatg*ctttatcaagcc-3′ (XbaI cleavage site, Stop codon underlined, histidine tag in italics) and cloned into mammalian expression vector pcDNA3.1(+) (Invitrogen, Carlsbad, CA, USA). The expression construct was transfected (FuGENE 6 Transfection Reagent, Roche) into HEK-293 cells (ATCC, CRL-1573), stable cell lines were selected in the presence of 500 µg/ml of Geneticin G418 antibiotic (Invitrogen), and recombinant protein, termed Tsp-C18, was purified from the conditioned media (CM) by metal ion affinity chromatography (ProBond Purification System; Invitrogen) and dialyzed against 1×PBS, pH 7.0. Tsp1-C18 was further purified by gel filtration using Superdex 200 column (GE Healthcare Life Sciences, Uppsala, Sweden) and separated fractions were analyzed by Western blot using anti-all-18 antibody (14). Combined fractions containing either high molecular weight (MW) or core Tsp1-C18 were digested with heparitinase I (Sigma Aldrich) (0.4–1,5 mU/µg of protein) in 50 mmol/l Tris-HCl, 50 mmol/l NaCl, 15 mM CaCl_2_, pH 7.0, at 37°C overnight. Fractions containing high MW Tsp1-C18 were also digested with chondroitinase ABC (Sigma Aldrich) (17 mU/µg of protein) in 50 mmol/l Tris-HCl, 50 mmol/l NaCl, pH 8.0, or with combination of both enzymes in 50 mmol/l Tris-HCl, 50 mmol/l NaCl, 15 mM CaCl_2_, pH 7.5, at 37°C overnight. Two fractions containing high MW Tsp1-C18 with GAG chains of variable lengths (fraction 29/Long GAG chain and 33/Intermediate-GAG-chain, see the result section for more information), as well as two apparently non-glycosylated core protein fractions (39 and 40 combined) were used for the subsequent binding and migration assays. In addition, an insert encoding for the full-length short isoform of collagen XVIII (Short-ColXVIII) was assembled from murine *Col18a1* cDNA clones [27]–[29], and cloned into the mammalian expression vector pREP7 (Invitrogen). The expression construct was transfected into 293-EBNA cells (Invitrogen), and stable cell lines were selected in the presence of 200 µg/ml of Hygromycin B (Invitrogen). Serum-free CM from the Short-XVIII (2) was collected for the binding and migration assays.

### Solid phase binding assay

Maxisorp 96-well plates (U96 from VWR International, Amsterdam, The Netherlands) were coated overnight with purified Tsp-C18 carrying GAG chains of variable lengths (Long GAG chain, Intermediate GAG chain and Core-Tsp1-C18) diluted 1∶10 with phosphate buffered saline (PBS), pH 7.5, as well as with the serum-free CM collected from 293-EBNA cells expressing the Short-ColXVIII. After washing with PBS- 0.05% Tween 20, the wells were blocked with 5% skimmed milk powder in PBS for 1 h. After washing, a dilution range of recombinant L-selectin-IgM chimera [30] or recombinant human MCP-1 (Peprotech EC) was added to the wells. L-selectin-IgM was detected with HRP-conjugated rabbit anti-human IgM (2 µg/ml; DAKO, Glostrup, Denmark) after repeated washes with PBS-Tween. MCP-1 was detected with monoclonal mouse anti-human MCP-1 antibody (0.5 µg/ml; eBioscience, CA, USA), followed by HRP-labeled goat anti-mouse immunoglobulins (DAKO, Glostrup, Denmark). HRP-conjugated secondary antibodies were detected with 3,3′,5,5′-tetramethylbenzidine substrate (Sigma, Zwijndrecht, The Netherlands) for 15 min in the dark, and the color reaction was quenched by H_2_SO_4_. Absorbance was measured at 450 nm with a microplate reader. All incubations were done at room temperature in a volume of 100 µl/well.

### Migration assay

Mouse leukemic monocyte/macrophage cell line (RAW 264.7) was purchased from ATCC (Wesel, Germany). Cells were cultured in DMEM containing 4.5 g/L glucose supplemented with 10% fetal bovine serum, 1% penicillin and streptomycin, and 1% L-glutamine (all purchased from Invitrogen). The migration assays were done using ChemoTx chemotaxis system according to the manufacturer’s protocol (NeuroProbe, Inc.; USA). In brief, the polycarbonate membranes (8 mm^2^ filter area; 3 µm pore size) were coated overnight at 4°C with albumin (control; 10 µg/ml; Sigma), heparin-albumin (10 µg/ml; Sigma), Long-GAG-chain, Intermediate-GAG-chain and Core-Tsp1-C18 (diluted 1∶4 with PBS), as well as with undiluted serum-free Short-ColXVIII CM. The membrane was washed with culture medium, and recombinant human MCP-1 (10 ng/ml in culture medium; Peprotech EC) was added to the lower chambers of the plate, and RAW 264.7 cells (1×10^4^) in 20 µl culture medium were added to the upper chamber. The plate was incubated in 37°C for 4 hours. The membrane was washed with PBS. The upper side of the membrane was cleaned with cotton swab and the membrane was fixed in 4% formaldehyde. The lower side of the membrane was stained with DAPI and the cells attached to the lower side were counted using a Leica DM 4000B immunofluorescence microscope. Meanwhile, the plate was centrifuged and the lower chambers of the plate were fixed with 4% formaldehyde. The cells in the lower chambers were also counted under the microscope as mentioned above. The total number of migrated cells in the control albumin-coated wells was set to 1, and the other values were calculated accordingly.

### Statistics

Statistical analysis was performed using the One-Way ANOVA followed by Dunnett post-hoc multiple comparison test in SPSS 20.0 software, with P<0.05 considered as statistically significant.

## Results

### Less renal function loss in collagen XV and XVIII deficient mice after I/R

**Figure 1:**
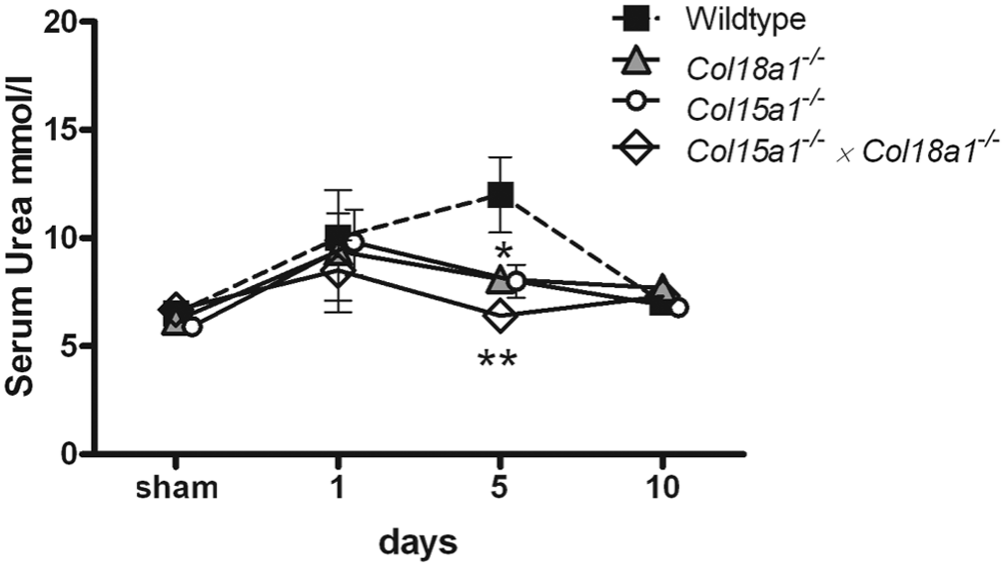
Serum urea levels in WT, collagen XV and/or XVIII deficient mice after renal I/R. WT mice showed an increased serum urea levels at day 5 after I/R while all collagen mutant mice (*Col15a1^−/−^* and *Col18a1^−/−^* p<0.05, and *Col15a1^−/−^×Col18a1^−/−^* p<0.01) had significantly lower serum urea levels compared to WT at this time point. The results are expresses as serum urea mmol/l ± SEM. *: p<0.05, **: p<0.01.

We evaluated renal function by measuring urea levels in serum samples collected at different time points after I/R, and compared them to those of sham operated mice at day 0. As expected, the WT mice showed a worsening in renal function manifested by a rise in serum urea level (highest value at day 5) which decreased to the baseline level at day 10 due to regeneration of renal cells (Fig. 1). There was no significant difference between mutant and WT mice at day 1 and day 10. However, 5 days after I/R both *Col18a1^−/−^* and *Col15a1^−/−^* mutants had significantly lower serum urea values compared to WT animals (both p<0.05). The decrease in serum urea was even more significant in *Col15a1^−/−^×Col18a1^−/−^* double mutant mice compared to WT controls (p<0.01; Fig. 1). These results indicate that our mutant animals have less renal function loss at day 5 than control WT mice.

### Less influx of inflammatory cells into collagen XV and XVIII deficient kidneys after I/R

**Figure 2:**
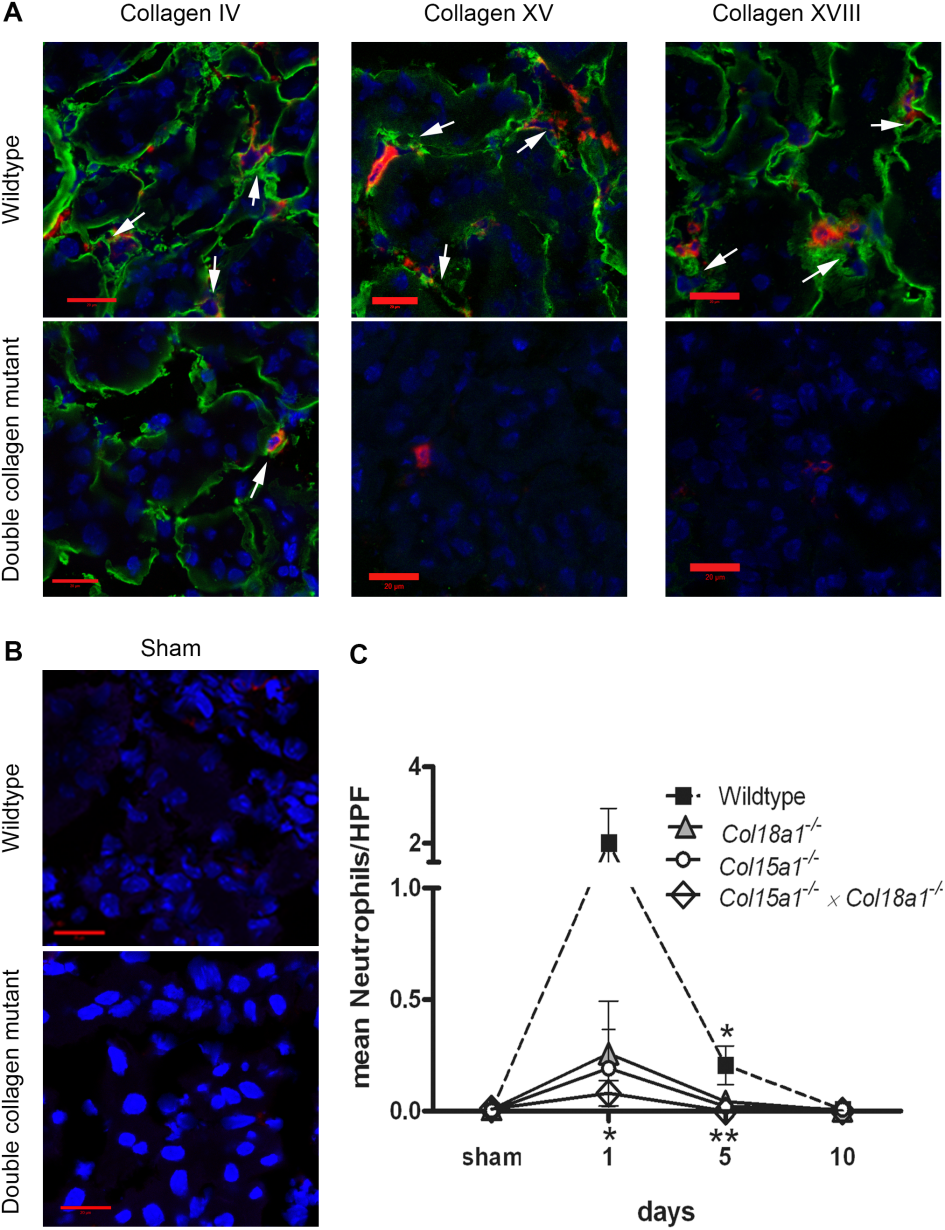
Reduced neutrophil influx after renal I/R in the mutant mice for collagen XV and XVIII. *A:* Immunofluorescent staining for collagen IV, XV and XVIII (green) and neutrophils (red) in WT and double mutant mice at day 1 after I/R showed presence of collagen IV, XV and XVIII in peritubular capillaries (white arrows) in WT mice and accumulation of neutrophils around these capillaries. Less neutrophil influx (in red) was observed in double mutant mice, also lacking collagen XV and XVIII signals (in green). Nuclei are shown in blue. Scale bars 20 µm. *B:* Immunofluorescent staining for neutrophils (red) and nuclei (blue) in WT and double mutant sham operated mice at day 1 after I/R showed the absence of neurophils in renal tissues. Scale bars 20 µm. *C:* Number of neutrophils per HPF (High Power Field) at different timepoints after I/R. At day 1 after reperfusion double mutant mice showed a decreased number of neutrophils compared to WT mice (p<0.05). At day 5 significantly less neutrophils were observed in kidneys of *Col15a1^−/−^* and *Col18a1^−/−^* mice compared to WT (p<0.05) as well as in those of double mutant mice compared to WT (p<0.01). Data is presented as mean ± SEM. *: p<0.05, **: p<0.01.

**Figure 3:**
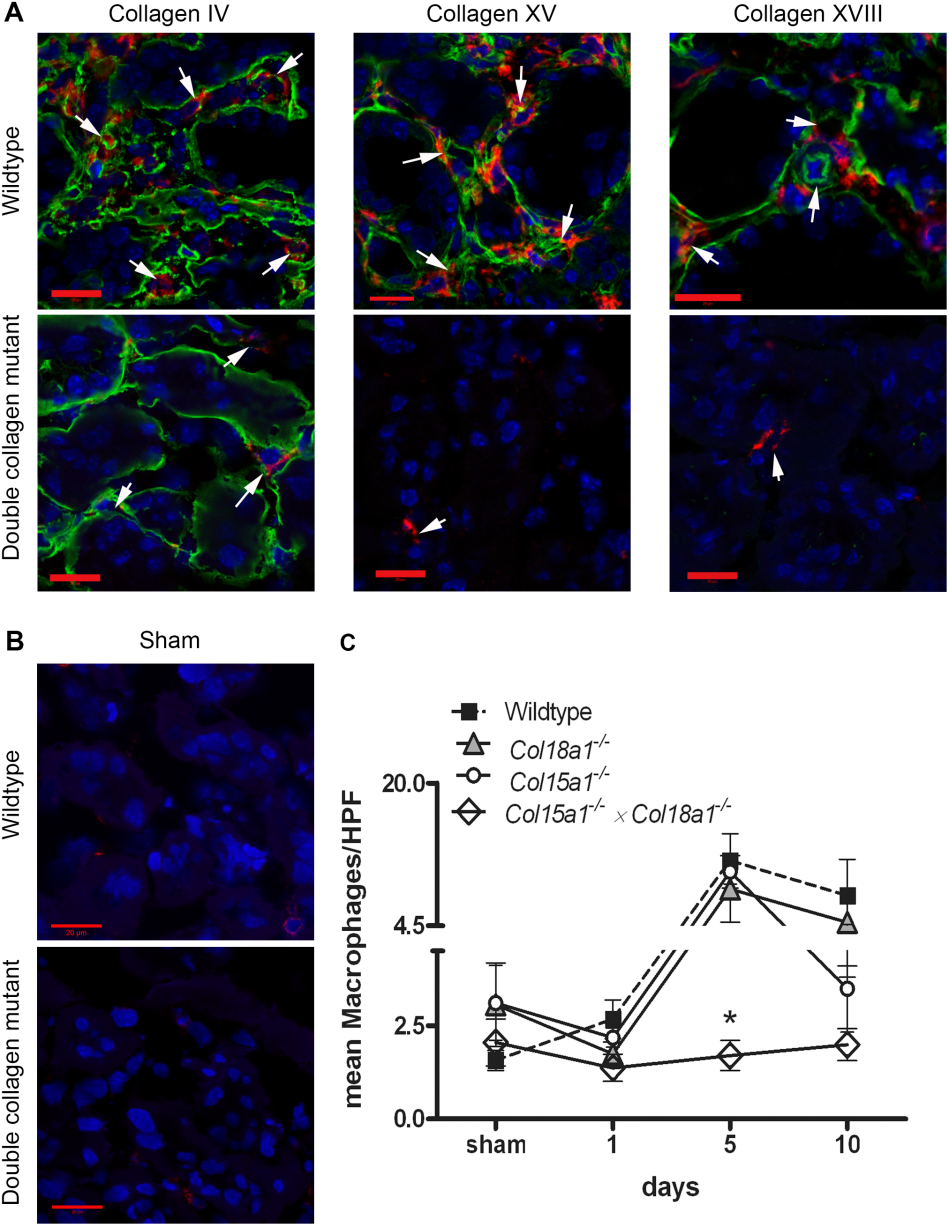
Reduced macrophage/monocyte influx after renal I/R in double mutant mice for collagen XV and XVIII. *A:* Immunofluorescent staining for collagen IV, XV and XVIII (green) and macrophages (red) in WT and double mutant mice at day 5 after I/R showed presence of collagen IV, XV and XVIII in peritubular capillaries (white arrows) in WT mice and accumulation of macrophage/monocytes around these capillaries. Less macrophage influx (in red) was observed in double mutant mice, also lacking collagen XV and XVIII signal (in green). The nuclei are stained in blue. Scale bars 20 µm. *B:* Immunofluorescent staining for macrophages (red) and nuclei (blue) in WT and double mutant sham operated mice at day 5 after I/R showed no macrophage/monocyte in renal tissues. Scale bars 20 µm. *C:* Number of macrophage/monocytes per HPF (High Power Field) at different timepoints after I/R. At day 5 significantly less macrophage/monocytes were observed in kidneys of *Col15a1^−/−^×Col18a1^−/−^* mice compared to WT (p<0.05). Data is presented as mean ± SEM. *: p<0.05.

It is known that I/R evokes a strong inflammatory reaction, characterized by an early neutrophil influx, followed by monocyte/macrophage recruitment. Immunofluorescent staining of kidney samples confirmed that collagen XV and XVIII localize in peritubular capillary and tubular BMs, analogously to a common BM marker collagen IV (Fig. 2A and 3A). Neutrophil recruitment was the highest at 1 day after I/R in WT mice (Fig. 2C). All collagen deficient groups showed lower numbers of neutrophils compared to WT group at day 1, but only in the *Col15a1^−/−^×Col18a1^−/−^* double mutant mice the difference was statistically significant (p<0.05). Nevertheless, at day 5 the neutrophil influx was significantly reduced in *Col18a1^−/−^* (p<0.05), *Col15a1^−/−^* (p<0.05) and *Col15a1^−/−^×Col18a1^−/−^* double deficient mice (p<0.01) compared to WT controls (Fig. 2A–C). Sham animals in WT group and double collagen deficient group showed no neutrophil influx (Fig. 2B). As expected, monocyte/macrophage influx peaked at day 5 after I/R injury and started to decline at day 10. In all time points *Col15a1^−/−^×Col18a1^−/−^* mice showed lower macrophage influx compared to WT mice (Fig. 3A–C); this difference was significant at day 5 after I/R (p<0.05; Fig. 3C). As shown in Fig. 3B no macrophage staining was found in WT and double collagen deficient sham animals (Fig. 3B). These data indicate impaired influx of inflammatory cells after renal I/R in mice lacking BM-associated collagens XV and XVIII.

### Less tubular injury and tubular activation in collagen XV and XVIII deficient kidneys after I/R

**Figure 4:**
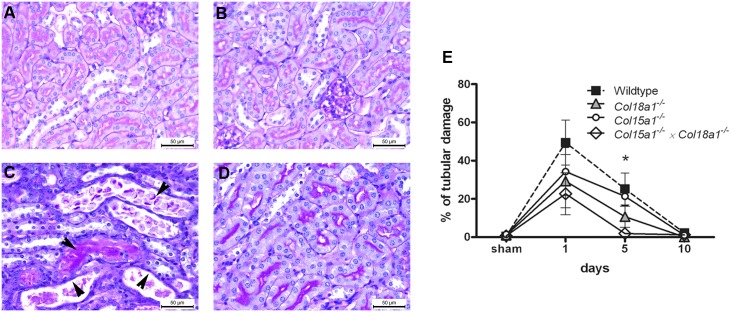
Tubular damage is reduced in double mutant mice deficient of collagen XV and XVIII compared to the WT mice. *A–D:* Paraffin-embedded sections stained with periodic acid Schiff's (PAS) reagent in sham operated WT(day 1; *A*), in sham-operated *Col15a1^−/−^×Col18a1^−/−^* compound mutant mice (day 1; *B*) and in WT (*C*) and *Col15a1^−/−^×Col18a1^−/−^* compound mutant mice (*D*) at day 5 after renal I/R. WT mice showed tubular casts, tubular widening and flattening, and loss of nuclei in tubular cells (black arrows). Such damage was not observed in double collagen mutant mice kidneys. Scale bars 50 µm. *E:* Percentage of tubular damage was determined at different time points after I/R (see methods) in the *Col15a1^−/−^*, *Col18a1^−/−^* and *Col15a1^−/−^×Col18a1^−/−^* double mutant mice compared to the WT controls (*: day 5 p<0.01 double mutant mice compare to WT). Data is presented as mean percentage ± SEM.

Tubular injury, determined as the percentage of necrotic tubules in kidney specimen, showed correlation with the inflammatory cell influx. One day after I/R all animals showed increased tubular injury. Although none of the single mutant groups was significantly different from WT, compound *Col15a1^−/−^×Col18a1^−/−^* mutants had the lowest amount of tubular injury. At day *5, Col15a1^−/−^×Col18a1^−/−^* double null mice showed also statistically significantly lower tubular damage compared to WT mice (p<0.05). At day 10, tubular injury was reduced in all animals to the level of sham values due to regeneration capability of tubular cells after I/R (Fig. 4E). No tubular injury was seen in sham-operated WT and double KO mice groups (Fig. 4A–B). Quantification of tubular injury in the single *Col15a1^−/−^* and *Col18a1^−/−^* mutant mice showed a tubular damage score in between the WT and double mutant mice. All together, the absence of BM-associated collagens XV and XVIII appears to lead to less severe tubular damage after I/R injury.

**Figure 5:**
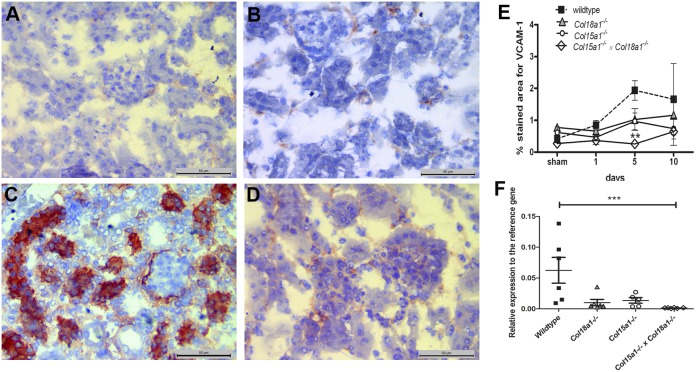
Tubular cell activation marker VCAM-1 is reduced in double mutant mice, deficient of collagen XV and XVIII compared to the WT mice. *A–D:* Cryosections stained for VCAM-1 expression in sham operated (day 1; *A*), in sham-operated *Col15a1^−/−^×Col18a1^−/−^* compound mutant mice (day 1; *B*) and in WT (*C*) and *Col15a1^−/−^×Col18a1^−/−^* compound mutant mice (*D*) at day 5. WT mice showed an upregulation of VCAM-1 in tubular compartment while sham and double collagen deficient mice showed a weak VCAM-1 expression in between of tubuli (peritubular capillaries). Scale bars 50 µm. *E:* Tubular cell activation was quantified as percentage of VCAM-1 expression in tubular cells at different timepoints after I/R in the *Col15a1^−/−^*, *Col18a1^−/−^ and Col15a1^−/−^×Col18a1^−/−^* double compound compared to WT mice (**: at day 5 p<0.01 double mutant mice compared to WT) (see methods). Data is presented as mean percentage ± SEM. *F:* Quantitative RT-PCR on RNA isolated from renal tissue, day 5 after I/R. VCAM-1 mRNA expression is reduced in all mutant mice, and reached statistical significance in the double KO mice (***: p<0.001).

Our results in WT mice showed upregulation of VCAM-1 in activated tubular cells upon I/R (Fig. 5C versus A (sham)). In the double mutant mice no tubular VCAM-1 expression was induced at day 5 compared to WT mice (Fig. 5E: p<0.01; Fig. 5D versus 5B). Sham-operated WT and double KO mice did only express low amounts of VCAM-1 in a peri-tubular fashion (Fig. 5A and B respectively). Single *Col15a1^−/−^* and *Col18a1^−/−^* mice showed a non-significant reduction in tubular cell activation on day 5 and 10 (Fig. 5E). Quantitative RT-PCR, done on RNA purified from day 5 kidneys corroborate the VCAM-1 protein staining (Fig. 5F). This indicates less tubular cell activation at this time point in the double collagen mutant mice which correlates with lower tubular damage score in these mice at day 5 after I/R.

### Inflammatory cytokines are upregulated in double collagen deficient mice

**Figure 6:**
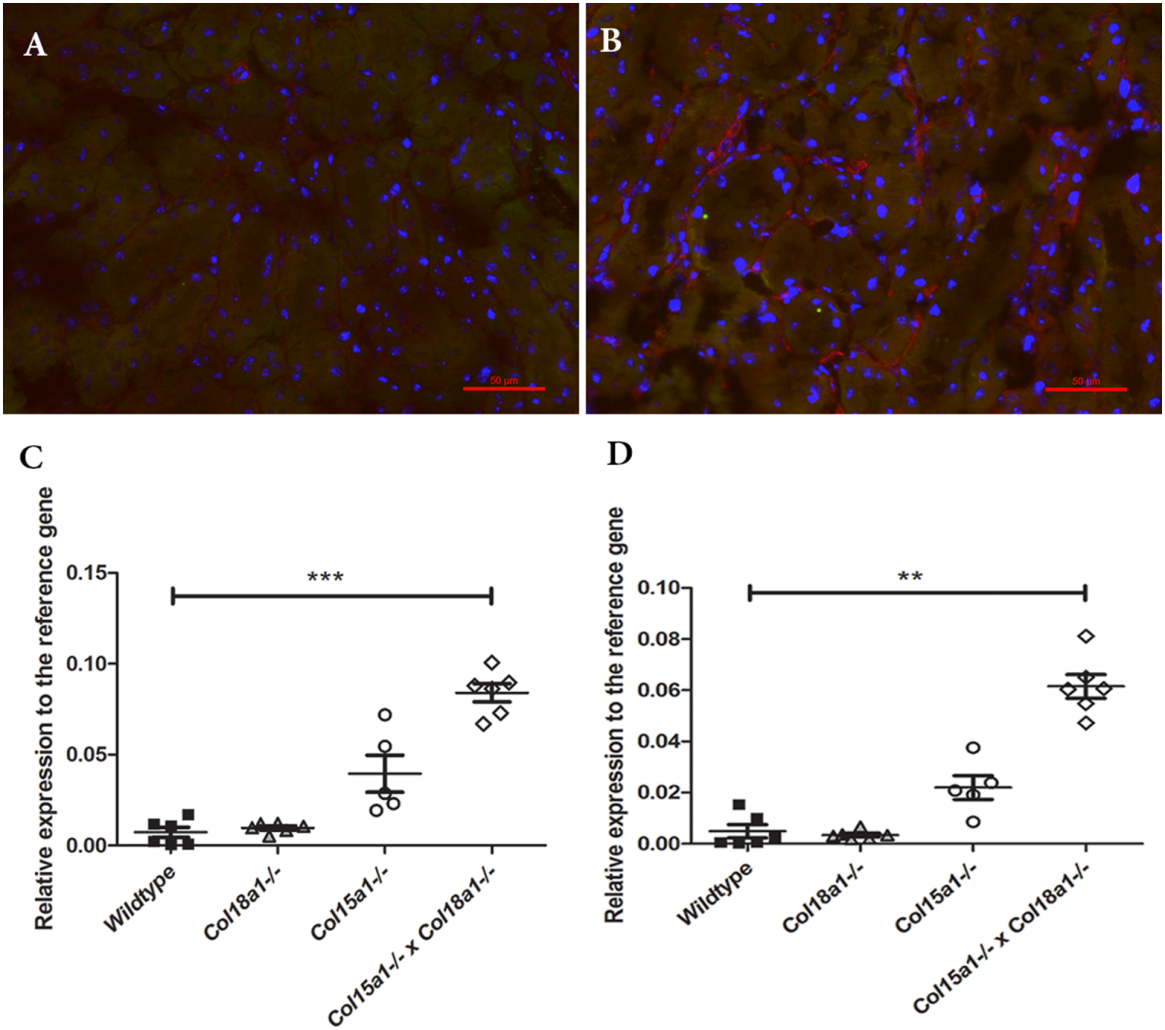
Increased expression of TNF-α and MCP-1 in double mutant mice, lacking both collagen XV and XVIII compared to WT. *A–B:* Immunofluorescent staining of MCP-1 (red) in WT (*A*) and double mutant mice (*B*) at day 5. Double mutant mice showed an increased expression of MCP-1 in peri-tubular capillaries. Scale bars 20 µm. *C:* mRNA expression of MCP-1 in renal tissue of *Col15a1^−/−^*, *Col18a1^−/−^ and Col15a1^−/−^×Col18a1^−/−^* double compound compared to WT mice at day 5 after I/R (***: p<0.001 double mutant mice compared to WT). *D:* mRNA expression of TNF-α in renal tissue of *Col15a1^−/−^*, *Col18a1^−/−^ and Col15a1^−/−^×Col18a1^−/−^* double compound compared to WT mice at day 5 after I/R (**: p<0.01 double mutant mice compared to WT).

To further investigate the mechanism behind the reduced influx of the inflammatory cells in the collagen mutant mice, we measured the gene expression of MCP-1 and TNF-α in all groups at day 5 (Fig. 6C–D). The result showed a significantly higher expression of both MCP-1 and TNF-α in double deficient mice at day 5 compared to WT mice (p<0.001 and p<0.01 respectively). The result for MCP-1 was confirmed by immunohistochemistry, showing more MCP-1 expression in peritubular capillaries of double deficient mice (Fig. 6A–B). Thus, despite increased expression of pro-inflammatory cytokines in the collagen XV and/or XVIII deficient mice, the reduced influx of inflammatory cells in the same animals underlines the importance of collagen XV and XVIII for leukocyte entry.

### Collagen XVIII proteoglycan is involved in monocyte/macrophage migration *in vitro*

**Figure 7:**
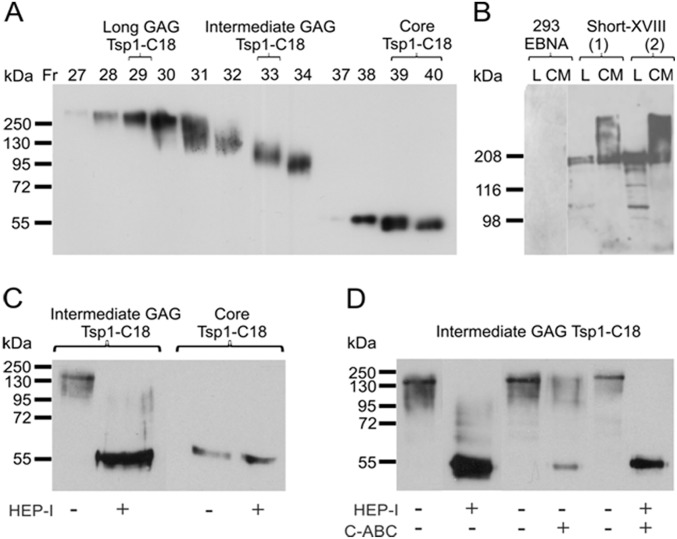
Production and characterization of recombinant mouse Tsp1-C18 and Short-XVIII by Western blotting with anti-all-18 antibody. *A:* Gel filtration fractions of recombinant Tsp1-C18 with variable degree of glycosylation and non-glycosylated core protein of ∼55 kDa separated by 10% SDS-PAGE. Fractions 29 and 33 with Long and Intermediate GAG chains, respectively, and 39–40 containing core Tsp-C18 were used for binding and migration experiments. *B:* Expression of full-length Short-XVIII by two representative stable 293-EBNA clones 1 and 2. Apparently non-glycosylated Short-XVIII of ∼180 kDa was detected in cell lysates (L). Conditioned cell culture media (CM) contained highly glycosylated recombinant Short-XVIII migrating as a smear above 200 kDa in 7% SDS-PAGE. Control 293-EBNA cell lysate and CM did not show reactivity for anti-all-18 antibody with short exposure. *C:* Heparitinase I treatment of intermediate Tsp1-C18 (fractions 31 and 33, MW ∼130 kDa) removed most GAG chains and revealed a core protein of ∼55 kDa. Heparitinase I treatment did not alter the size of low MW Tsp1-C18 (fractions 39–40) suggesting that this band represents non-glycosylated core protein. *D:* Heparitinase I and chondroitinase ABC treatments of intermediate Tsp1-C18 (fractions 31 and 33) indicate that most of the GAGs within recombinant Tsp1-C18 produced in HEK-293 cells are HS chains.

N-terminal non-collagenous portion of short collagen XVIII, Tsp1-C18, was expressed in mammalian HEK-293 cells and purified from CM using metal ion affinity and gel filtration chromatography. Western blot analysis of collected fractions with anti-all-18 antibody revealed that separated fractions contained GAG chains with variable lengths (Fig. 7A). Combined fractions containing intermediate or low molecular weight (MW) Tsp1-C18 were digested with heparitinase I. Enzyme treatment of intermediate MW (∼130 kDa) Tsp1-C18 removed most of the GAG chains, reduced the size of the protein smear to 70–100 kDa, and resulted in the appearance of a ∼55 kDa protein band (Fig. 7C). No apparent change in the molecule size was seen when low MW protein was treated with heparitinase I suggesting that the ∼55 kDa band represents the core Tsp1-C18 (Fig. 7C). Thus, the gel filtration chromatography allowed separation of recombinant Tsp1-C18 fractions with variable degree of glycosylation and non-glycosylated core protein. Besides heparitinase I alone, the intermediate MW Tsp1-C18 was digested also with chondroitinase ABC or with combination of both enzymes. Heparitinase I plus chondroitinase treatment totally abolished the protein smear and revealed the ∼55 kDa core protein (Fig. 7D). Chondroitinase treatment alone had a minor effect on Tsp1-C18 resulting in the appearance a weak core band while most of the protein remained glycosylated with no apparent change in the molecule size (Fig. 7D). These data indicate that the N-terminal portion of short collagen XVIII produced in HEK-293 cells carries GAGs and most of them are HS chains.

The full-length Short-XVIII protein was detected in the stable 293-EBNA cell lysate and CM with mouse anti-all-18 antibody (Fig. 7B). In the CM this antibody recognized a smear with MW well above 200 kDa indicating the presence of GAG chains, while in the cell lysate a protein with MW of ∼180 kDa was detected. The latter closely corresponds to the size of non-glycosylated core protein deduced form the amino acid sequence of the mouse short collagen XVIII isoform, as well as that of human, rat and chicken short isoform (6, 31, 32).

**Figure 8:**
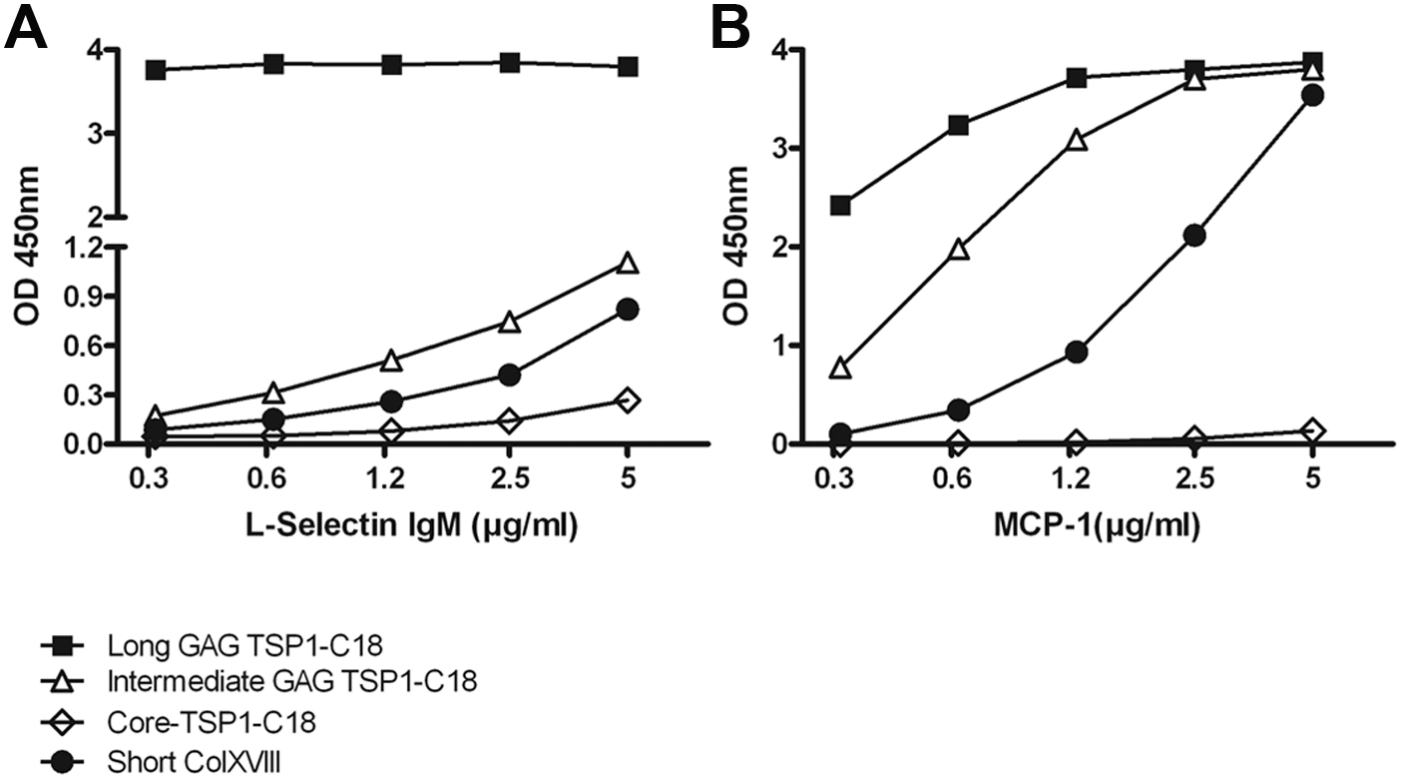
Collagen XVIII interacts with L-selectin and MCP-1 via its N-terminal HS side chains. *A:* Solid phase binding assay showed a dose dependent binding of L-selectin to full-length short collagen XVIII (Short-XVIII), and to its N terminal non-collagenous portion (Tsp1-C18) bearing HS side chains. The interaction of L-selectin with HS side chains on collagen XVIII is stronger when the HS side chains are longer. *B:* The interaction of MCP-1 with Short-XVIII and Tsp1-C18 bearing HS side chains in solid phase binding assay. MCP-1 interacts with collagen XVIII in a dose-dependent manner and the interaction is dependent on the length of HS side chains. Both assays were performed at least three times and the graphs show a representative experiment. PBS coated wells were used as negative controls (not shown).

We performed solid phase binding experiments to test the interaction of collagen XVIII with L-selectin and MCP-1. First, to verify the direct interaction between HS/CS side chains within the short collagen XVIII isoform and L-selectin, recombinant human L-selectin-IgM chimeric protein was incubated in wells coated either with the CM containing full length short isoform (Short-XVIII), or with purified N-terminal Tsp1-C18 fragments of this isoform with various degrees of glycanation. The results showed a dose-dependent binding of L-selectin to Short-XVIII. Moreover, the binding of L-selectin to high MW Tsp1-C18 with the longest GAG chains was very strong even at the lowest L-selectin concentrations. No binding was detected between L-selectin and the low MW Tsp1-C18 which lacked GAG chains (Fig. 7A, C), and moderate binding was observed to the fragment with intermediate GAG chains (Fig. 8A), thus confirming the view that GAG chains have a role in L-selectin binding.

In order to test the binding of collagen XVIII to MCP-1, a similar solid phase binding assay was done by incubating increasing concentrations of MCP-1 on collagen XVIII coated wells. As it is shown in Fig. 8B, the Tsp1-C18 fragment lacking GAG chains showed no interaction with MCP-1, while MCP-1 bound dose-dependently to the full length molecule and to the two N-terminal fragments carrying intermediate and long GAG chains. The high MW Tsp1-C18 showed the strongest interaction with MCP-1 (Fig. 8B). These data suggests that collagen XVIII HS/CS side chains are involved in influx of monocytes mostly via interaction with MCP-1 and/or L-selectin.

**Figure 9:**
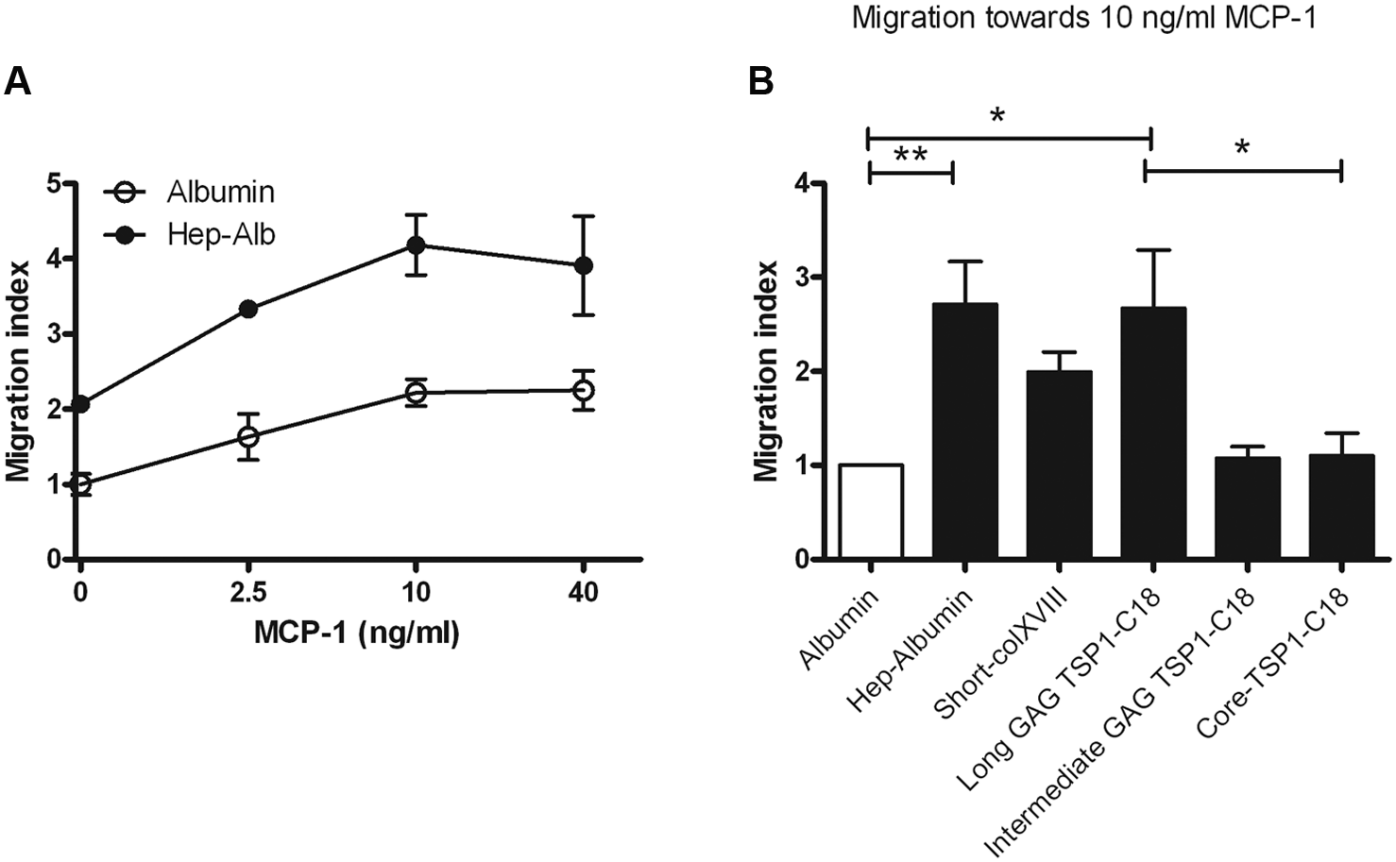
MCP-1-induced monocyte migration is increased in the presence of immobilized heparin-albumin and glycosylated collagen XVIII. *A:* MCP-1 dose dependently increased the migration of monocytes over a porous membrane. Immobilization of heparin-albumin, mimicking an artificial BM HSPG, promotes monocyte transmigration. Spontaneous migration over albumin-coated membrane in the absence of MCP-1 was set as 1 and the other values were calculated accordingly. The error bars represent SEM. *B:* Transmigration of monocytes towards MCP-1 (10 ng/ml) was increased in the presence of heparin-albumin and collagen XVIII with long GAG chains. Heparin-albumin increased the monocyte migration significantly compared to albumin coated membrane (p<0.01). N-terminal fragment of short collagen XVIII with long GAG chains promoted transmigration significantly compared to albumin and N-terminal fragment without GAG chain (both p<0.05). Relative to albumin, also the full-length short collagen XVIII promoted MCP-1-induced monocyte transmigration to some extent (not significant). Data is calculated relative to migration over albumin-coated membrane towards 10 ng/ml MCP-1. The error bars represent SEM. *: p<0.05, **: p<0.01.

We next investigated the involvement of HS/CS side chains of collagen XVIII in leukocyte migration using an *in vitro* approach. To this end, a Transwell migration assay was performed in which mouse leukemic monocyte macrophage RAW 264.7 cells were induced to migrate towards MCP-1 over porous membranes coated with albumin, heparin-albumin, Short-XVIII or N-terminal non-collagenous fragments with differing GAG chain lengths. The filter-immobilized heparin-albumin is a model compound that represents here a BM HSPG. As expected, adding MCP-1 to the lower compartment of albumin-coated control wells resulted in a dose-dependent increase in migration of the monocytes (Fig. 9A). Coating with heparin-albumin raised the amount of spontaneous and MCP-1-induced cell migration in comparison to albumin. In addition, in the presence of 10 ng/ml MCP-1 the number of migrated cells through filters coated with heparin-albumin (p<0.01) or with Tsp1-C18 carrying the longest GAG chains (p<0.05) raised significantly compared to their migration on albumin coated filters. Besides, RAW 264.7 cell migration was significantly increased in the presence of Tsp1-C18 with long GAG chains by comparison to core protein (p<0.05). Also full-length Short-XVIII increased the migration compared to albumin or core Tsp1-C18, but the differences were not statistically significant. These *in vitro* data indicate that GAG polysaccharide side chains in the tested artificial BMs substantially contribute to chemokine-driven/induced transmigration of monocytes.

## Discussion

We demonstrate here that relative to the WT controls, the mice lacking expression of BM-associated collagen types XV or XVIII, and in particular mice lacking both BM-associated collagens *(Col15a1^−/−^×Col18a1^−/−^* compound mutant), show less susceptibility to tubular damage and loss of kidney function after renal I/R. Despite an upregulation of inflammatory cytokines MCP-1 and TNF-α, we observed less inflammatory cell influx at day 5 after I/R, and less tubular cell activation and less tubular damage on the same day. These mice also have a better renal function at day 5, demonstrated by lower urea levels in the circulation. Complementing *in vitro* data show that the GAG polysaccharide side chains of the short collagen XVIII isoform are involved in the binding of L-selectin and MCP-1, facilitating the migration of leukocytes into the inflamed kidney. Together, these data suggest an important role for collagen XV and XVIII in inflammation-induced renal damage after I/R.

Tubular damage in I/R model has been shown to be directly related to leukocyte influx [22]. Besides, infiltrated leukocytes, especially monocytes/macrophages contribute to epithelial to mesenchymal transition of the tubular epithelial cells, which upon de-differentiation become ICAM-1/VCAM-1 positive [31]. This tubular expression of adhesion molecules is not involved in leukocyte influx, since this is predominantly orchestrated at the level of the endothelial cells, rather form a retention motif for VLA-4 positive leukocytes and can be used as a measure for tubular activation/de-differentiation [32]. Our compound mutant mice have a better renal function and less tubular damage, most likely due to less inflammatory cell influx into diseased kidney. Therefore, based on our novel and previous findings we consider three different mechanisms underlying the reduced leukocyte recruitment after I/R in the *Col15a1^−/−^×Col18a1^−/−^* mice.

First, we reported broadened tubular BM in *Col18a1^−/−^* mice compared to WT [33], suggesting that a modified physical structure of BM in our mutant mice could be partially responsible for less leukocyte influx after I/R. Kinnunen *et al.* showed that although it is not noticeable in normal condition, the tubular BM of collagen XVIII deficient mice shows some structural abnormalities [16] which might result in an altered response after I/R and affect leukocyte influx and the degree of damage. However, that needs further research to be confirmed.

Second, Celie *et al.* [21] previously showed that BM proteoglycans, including collagen XVIII, can bind to L-selectin and facilitate leukocyte migration. Kawashima *et al*. suggested that collagen XVIII may provide a link between selectin-mediated cell adhesion and chemokine-induced cellular activation and accelerate the progression of leukocyte infiltration in renal inflammation. They also demonstrated a direct interaction of collagen XVIII HS side chains with L-selectin and MCP-1 *in vitro* [34]. Others showed the interaction of proteoglycans with the leukocyte adhesion molecules MAC-1 and VLA-4 [35], [36]. We confirmed here the direct interaction between L-selectin and the tubular BM-specific short collagen XVIII molecule. Furthermore, we showed that the long HS chains within N-terminal non-collagenous portion of this particular isoform contain HS domains that can interact with L-selectin. This interaction indicates the involvement of collagen XVIII GAG chains in leukocyte adhesion and migration via interaction with L-selectin, and can at least partly explain less influx of inflammatory cells in collagen XVIII deficient mice.

Third, proteoglycans are known to stabilize gradients of chemokines and cytokines [37], [38]. As shown by Celie *et al.,* binding sites for MCP-1, a potent chemoattractant for monocytes/macrophages, were increased after I/R and are predominantly mediated by HSPGs in BM [21]. Our data confirms the involvement of collagen XVIII GAG side chains in chemokine-derived leukocyte migration and solid phase binding assay showed the binding of collagen XVIII to MCP-1 *via* its GAG chains. The binding appeared to be stronger when the GAG side chains attached to collagen XVIII were longer indicating that certain HS domains/length of collagen XVIII are needed to efficiently bind this chemokine. Moreover involvement of specific GAG side chains of collagen XVIII in chemokine-induced leukocyte migration was confirmed by the significant increase in monocyte migration over filters coated with N-terminal fragment with longest GAG chains compared to filters coated with N-terminal fragments without GAG chains. Since the Coll XVIII Tsp1-C18 fragment has one predicted GAG attachment site, we assume that fraction 32–34 of the collagen XVIII Tsp1-C18 fragment (see Fig. 7A) has HS-GAG chains of intermediate length. However, we cannot exclude the possibility that these fractions with shorter HS-GAG side chain were decorated with different sulfation pattern that will influence L-selectin and MCP-1 binding and leukocyte migration. We show an increase in expression of MCP-1 in renal tissue of double collagen deficient mice at the same timepoint that the animals show the lowest influx of the cells. Our results show that even increased expression of MCP-1 does not lead to higher inflammatory cells influx if the BM HSPGs are absent. These findings highlight the importance of the interaction between collagen XV and XVIII and chemokines such as MCP-1 in cell influx.

Since the two BM collagens type XV and XVIII have some similarities, including the Tsp-1 domain at the N-terminus and the GAG side chains, we speculate that lacking both of those BM zone collagen/proteoglycan hybrid molecules results in a pronounced effect as shown by the lowest neutrophil and monocyte/macrophage influx and the highest expression of MCP-1 in double mutant mice in our study. Unfortunately, we could not test here the role of GAG chains of collagen XV, owing to the fact that our recombinant collagen XV is produced in insect cells and lack GAG chains [39]. The interactions of GAGs within collagen XV with L-selectin and MCP-1 have to be demonstrated and studied in detail to achieve full understanding of the roles of this BM collagen in kidney injuries.

Collectively, our data show that collagens XV and/or XVIII HSPGs can, *via* their GAG side chains, mediate leukocyte migration over vascular BMs. This novel functions of BM collagen XV and XVIII might operate besides to the described roles of laminin 411 and the BM low expression regions in leukocyte migration over BMs [40], [41]. Based on our results we speculate on new therapeutic interventions with (non-anticoagulant) heparinoids mimicking the BM collagens XV and XVIII GAGs after renal I/R and renal transplantation. Further experiments might prove the efficacy of this heparinoid intervention approach.

## References

[1] Kruegel J, Miosge N (2010) Basement membrane components are key players in specialized extracellular matrices. Cell Mol Life Sci 67: 2879–2895. 20428923 10.1007/s00018-010-0367-x PMC2921489

[2] Yurchenco PD, Patton BL (2009) Developmental and pathogenic mechanisms of basement membrane assembly. Curr Pharm Des 15: 1277–1294. 19355968 10.2174/138161209787846766 PMC2978668

[3] Kalluri R (2003) Basement membranes: Structure, assembly and role in tumour angiogenesis. Nat Rev Cancer 3: 422–433. 12778132 10.1038/nrc1094

[4] Amenta PS, Scivoletti NA, Newman MD, Sciancalepore JP, Li D, et al (2005) Proteoglycan-collagen XV in human tissues is seen linking banded collagen fibers subjacent to the basement membrane. J Histochem Cytochem 53: 165–176. 15684329 10.1369/jhc.4A6376.2005

[5] Ortega N, Werb Z (2002) New functional roles for non-collagenous domains of basement membrane collagens. J Cell Sci 115: 4201–4214. 12376553 10.1242/jcs.00106 PMC2789001

[6] Saarela J, Rehn M, Oikarinen A, Autio-Harmainen H, Pihlajaniemi T (1998) The short and long forms of type XVIII collagen show clear tissue specificities in their expression and location in basement membrane zones in humans. Am J Pathol 153: 611–626. 9708820 10.1016/S0002-9440(10)65603-9 PMC1852992

[7] Seppinen L, Pihlajaniemi T (2011) The multiple functions of collagen XVIII in development and disease. Matrix Biol 30: 83–92. 21163348 10.1016/j.matbio.2010.11.001

[8] Marneros AG, Olsen BR (2005) Physiological role of collagen XVIII and endostatin. FASEB J 19: 716–728. 15857886 10.1096/fj.04-2134rev

[9] Ramchandran R, Dhanabal M, Volk R, Waterman MJ, Segal M, et al (1999) Antiangiogenic activity of restin, NC10 domain of human collagen XV: Comparison to endostatin. Biochem Biophys Res Commun 255: 735–739. 10049780 10.1006/bbrc.1999.0248

[10] Sertie AL, Sossi V, Camargo AA, Zatz M, Brahe C, et al (2000) Collagen XVIII, containing an endogenous inhibitor of angiogenesis and tumor growth, plays a critical role in the maintenance of retinal structure and in neural tube closure (knobloch syndrome). Hum Mol Genet 9: 2051–2058. 10942434 10.1093/hmg/9.13.2051

[11] Fukai N, Eklund L, Marneros AG, Oh SP, Keene DR, et al (2002) Lack of collagen XVIII/endostatin results in eye abnormalities. EMBO J 21: 1535–1544. 11927538 10.1093/emboj/21.7.1535 PMC125362

[12] Eklund L, Piuhola J, Komulainen J, Sormunen R, Ongvarrasopone C, et al (2001) Lack of type XV collagen causes a skeletal myopathy and cardiovascular defects in mice. Proc Natl Acad Sci U S A 98: 1194–1199. 11158616 10.1073/pnas.031444798 PMC14731

[13] Rasi K, Piuhola J, Czabanka M, Sormunen R, Ilves M, et al (2010) Collagen XV is necessary for modeling of the extracellular matrix and its deficiency predisposes to cardiomyopathy. Circ Res 107: 1241–1252. 20847313 10.1161/CIRCRESAHA.110.222133

[14] Momota R, Naito I, Ninomiya Y, Ohtsuka A (2011) Drosophila type XV/XVIII collagen, mp, is involved in wingless distribution. Matrix Biol 30: 258–266. 21477650 10.1016/j.matbio.2011.03.008

[15] Meyer F, Moussian B (2009) Drosophila multiplexin (dmp) modulates motor axon pathfinding accuracy. Dev Growth Differ 51: 483–498. 19469789 10.1111/j.1440-169X.2009.01111.x

[16] Kinnunen AI, Sormunen R, Elamaa H, Seppinen L, Miller RT, et al (2011) Lack of collagen XVIII long isoforms affects kidney podocytes, whereas the short form is needed in the proximal tubular basement membrane. J Biol Chem 286: 7755–7764. 21193414 10.1074/jbc.M110.166132 PMC3048663

[17] Hagg PM, Hagg PO, Peltonen S, Autio-Harmainen H, Pihlajaniemi T (1997) Location of type XV collagen in human tissues and its accumulation in the interstitial matrix of the fibrotic kidney. Am J Pathol 150: 2075–2086. 9176399 PMC1858337

[18] Bellini MH, Coutinho EL, Filgueiras TC, Maciel TT, Schor N (2007) Endostatin expression in the murine model of ischaemia/reperfusion-induced acute renal failure. Nephrology (Carlton) 12: 459–465. 17803469 10.1111/j.1440-1797.2007.00850.x

[19] Hamano Y, Okude T, Shirai R, Sato I, Kimura R, et al (2010) Lack of collagen XVIII/endostatin exacerbates immune-mediated glomerulonephritis. J Am Soc Nephrol 21: 1445–1455. 20616167 10.1681/ASN.2009050492 PMC3013523

[20] Rienstra H, Katta K, Celie JW, van Goor H, Navis G, et al (2010) Differential expression of proteoglycans in tissue remodeling and lymphangiogenesis after experimental renal transplantation in rats. PLoS One 5: e9095. 20140097 10.1371/journal.pone.0009095 PMC2816722

[21] Celie JW, Rutjes NW, Keuning ED, Soininen R, Heljasvaara R, et al (2007) Subendothelial heparan sulfate proteoglycans become major L-selectin and monocyte chemoattractant protein-1 ligands upon renal ischemia/reperfusion. Am J Pathol 170: 1865–1878. 17525255 10.2353/ajpath.2007.070061 PMC1899444

[22] Jang HR, Ko GJ, Wasowska BA, Rabb H (2009) The interaction between ischemia-reperfusion and immune responses in the kidney. J Mol Med (Berl) 87: 859–864. 19562316 10.1007/s00109-009-0491-y

[23] Petrini G, Ochoa EJ, Serra E, Torres AM, Elias MM (2002) Fibronectin expression in proximal tubules from ischemic rat kidneys without reperfusion. Mol Cell Biochem 241: 21–27. 12482021 10.1023/a:1020878919459

[24] Wen X, Murugan R, Peng Z, Kellum JA (2010) Pathophysiology of acute kidney injury: A new perspective. Contrib Nephrol 165: 39–45. 20427954 10.1159/000313743

[25] Bonventre JV, Yang L (2011) Cellular pathophysiology of ischemic acute kidney injury. J Clin Invest 121: 4210–4221. 22045571 10.1172/JCI45161 PMC3204829

[26] Ylikarppa R, Eklund L, Sormunen R, Muona A, Fukai N, et al (2003) Double knockout mice reveal a lack of major functional compensation between collagens XV and XVIII. Matrix Biol 22: 443–448. 14614990 10.1016/s0945-053x(03)00074-x

[27] Rehn M, Pihlajaniemi T (1994) Alpha 1(XVIII), a collagen chain with frequent interruptions in the collagenous sequence, a distinct tissue distribution, and homology with type XV collagen. Proc Natl Acad Sci U S A 91: 4234–4238. 8183894 10.1073/pnas.91.10.4234 PMC43759

[28] Rehn M, Pihlajaniemi T (1995) Identification of three N-terminal ends of type XVIII collagen chains and tissue-specific differences in the expression of the corresponding transcripts. the longest form contains a novel motif homologous to rat and drosophila frizzled proteins. J Biol Chem 270: 4705–4711. 7876242 10.1074/jbc.270.9.4705

[29] Rehn M, Hintikka E, Pihlajaniemi T (1994) Primary structure of the alpha 1 chain of mouse type XVIII collagen, partial structure of the corresponding gene, and comparison of the alpha 1(XVIII) chain with its homologue, the alpha 1(XV) collagen chain. J Biol Chem 269: 13929–13935. 8188673

[30] Celie JW, Keuning ED, Beelen RH, Drager AM, Zweegman S, et al (2005) Identification of L-selectin binding heparan sulfates attached to collagen type XVIII. J Biol Chem 280: 26965–26973. 15917223 10.1074/jbc.M502188200

[31] Li Q, Liu BC, Lv LL, Ma KL, Zhang XL, et al (2011) Monocytes induce proximal tubular epithelial-mesenchymal transition through NF-kappa B dependent upregulation of ICAM-1. J Cell Biochem 112: 1585–1592. 21344487 10.1002/jcb.23074

[32] Oertli B, Beck-Schimmer B, Fan X, Wuthrich RP (1998) Mechanisms of hyaluronan-induced up-regulation of ICAM-1 and VCAM-1 expression by murine kidney tubular epithelial cells: Hyaluronan triggers cell adhesion molecule expression through a mechanism involving activation of nuclear factor-kappa B and activating protein-1. J Immunol 161: 3431–3437. 9759861

[33] Utriainen A, Sormunen R, Kettunen M, Carvalhaes LS, Sajanti E, et al (2004) Structurally altered basement membranes and hydrocephalus in a type XVIII collagen deficient mouse line. Hum Mol Genet 13: 2089–2099. 15254016 10.1093/hmg/ddh213

[34] Kawashima H, Watanabe N, Hirose M, Sun X, Atarashi K, et al (2003) Collagen XVIII, a basement membrane heparan sulfate proteoglycan, interacts with L-selectin and monocyte chemoattractant protein-1. J Biol Chem 278: 13069–13076. 12556525 10.1074/jbc.M212244200

[35] Zen K, Parkos CA (2003) Leukocyte-epithelial interactions. Curr Opin Cell Biol 15: 557–564. 14519390 10.1016/s0955-0674(03)00103-0

[36] Schlesinger M, Simonis D, Schmitz P, Fritzsche J, Bendas G (2009) Binding between heparin and the integrin VLA-4. Thromb Haemost 102: 816–822. 19888514 10.1160/TH09-01-0061

[37] Erickson AC, Couchman JR (2000) Still more complexity in mammalian basement membranes. J Histochem Cytochem 48: 1291–1306. 10990484 10.1177/002215540004801001

[38] Hynes RO (2009) The extracellular matrix: Not just pretty fibrils. Science 326: 1216–1219. 19965464 10.1126/science.1176009 PMC3536535

[39] Hurskainen M, Ruggiero F, Hagg P, Pihlajaniemi T, Huhtala P (2010) Recombinant human collagen XV regulates cell adhesion and migration. J Biol Chem 285: 5258–5265. 20040604 10.1074/jbc.M109.033787 PMC2820754

[40] Voisin MB, Probstl D, Nourshargh S (2010) Venular basement membranes ubiquitously express matrix protein low-expression regions: Characterization in multiple tissues and remodeling during inflammation. Am J Pathol 176: 482–495. 20008148 10.2353/ajpath.2010.090510 PMC2797906

[41] Korpos E, Wu C, Song J, Hallmann R, Sorokin L (2010) Role of the extracellular matrix in lymphocyte migration. Cell Tissue Res 339: 47–57. 19697064 10.1007/s00441-009-0853-3

